# MiR-30a-5p promotes cholangiocarcinoma cell proliferation through targeting SOCS3

**DOI:** 10.7150/jca.41437

**Published:** 2020-03-26

**Authors:** Jia Wei Zhang, Xing Wang, Gao Chao Li, Dong Wang, Sheng Han, Yao Dong Zhang, Chen Huan Luo, Hong Wei Wang, Wang Jie Jiang, Chang Xian Li, Xiang Cheng Li

**Affiliations:** Hepatobiliary Center, The First Affiliated Hospital of Nanjing Medical University, Key Laboratory of Living Donor Liver Transplantation, Nanjing, Jiangsu Province, China

**Keywords:** miR-30a-5p, cholangiocarcinoma, SOCS3, proliferation, apoptosis

## Abstract

**Background**: MicroRNAs (miRNAs) play important roles in the occurrence and development of cancers. In this project, we aimed to explore the role and molecular mechanism of mir-30a-5p in cholangiocarcinoma (CCA).

**Materials and Methods**: The expression profile and clinical significance of miR-30a-5p in CCA patients were investigated in 31 ICC and 52 ECC patients respectively. The role and mechanism of miR-30a-5p in CCA cells were investigated by up-regulating and inhibiting miR-30a-5p expression *in vitro* functional study.

**Results**: The expression of miR-30a-5p was increased in both CCA tissues and cells. The inhibition of miR-30a-5p decreased cell proliferation and induced cell apoptosis while overexpression of miR-30a-5p achieved the opposite effect. Furthermore, SOCS3 was down-regulated in ICC and ECC tissues and negatively regulated by miR-30a-5p. Dual-luciferase reporter assay revealed that co-transfection of miR-30a-5p significantly inhibited the activity of firefly luciferase reporter carrying the wild-type 3′UTR of SOCS3. The inhibition of SOCS3 could largely rescue the inhibitory effect of miR-30a-5p inhibition on CCA cells proliferation. In clinical, up-regulated miR-30a-5p expression was correlated with large tumor size in both ICC and ECC cohorts.

**Conclusions**: miR-30a-5p promoted CCA cells proliferation through targeting SOCS3. These findings suggested that miR-30a-5p could be a potential therapeutic target.

## Introduction

Cholangiocarcinoma (CCA) is a highly malignant tumor originating from the bile duct epithelium, which can be categorized as intrahepatic cholangiocarcinoma (ICC), extra cholangiocarcinoma (ECC) according to the anatomical location. CCA is the second most frequent primary hepatobiliary malignancy and its incidence and mortality gradually increase in the last three decades 1, 2. Surgical resection is the only possible curative treatment for CCA patients, however, the rates of resectability and the long-term outcome after these therapies are less than satisfactory because of the high post-surgical recurrence 3. Therefore, elucidating the molecular mechanisms of CCA development and progression are urgently necessary to develop more efficient therapies for CCA.

MicroRNAs (miRNAs) are small non-coding RNAs of 20-22 nucleotides, which can silence or inhibit genes by binding to the target sites of messenger RNAs 4. It is reported that miRNAs are involved in multiple cellular processes, including cell growth, apoptosis, movement, and differentiation. More evidence showed that the dysfunction or abnormal expression of miRNAs maybe lead to incidence of human malignancies, and play important roles in tumor growth and metastasis^5^, ^6^. Furthermore, miRNAs can as the biomarker of the diagnosis and as potential cancer therapeutics 7, 8. Recently, increasing researches have revealed that miRNAs were powerful regulators in the tumorigenesis of CCA^9^, ^10^. The levels of miR‐433 and miR‐22 were down-regulated in CCA, and restoration of these miRNAs contributed to ciliary restoration and decreased the malignant phenotype of cancer cells by targeting histone deacetylase 6 9. Furthermore, miR‐21 was highly expressed in CCA and appeared to mediate resistance of CCA cells to HSP90 inhibitors by reducing levels of DNAJB5^10^.

The miR-30 family, which contains five members of distinct pre-mature miRNAs (miR-30a, -30b, -30c, -30d, -30e), has been proved to play diverse roles in regulating essential aspects of tumorigenesis, metastasis, chemo-resistance and clinical prognosis in several types of human cancers 11, 12. A large number of reports have demonstrated that miR-30a-5p, as a member of the mir-30 family, was down-regulated and associated with tumorigenesis and progression of prostate cancer, hepatocellular carcinoma, breast tumor and some other cancers 13-15. However, it has been reported that overexpression of miR-30a-5p contributed to initiation and progression of some tumors 12, 16. However, the expression and role of miR-30a-5p in CCA progression remain unclear.

In the current study, we aimed to explore the role and molecular mechanism of mir-30a-5p in CCA. Firstly, the expression profile of mir-30a-5p was detected in 83 CCA patients, and analyzed the association with clinical characteristics and prognosis of patients. Subsequently, we investigated the functional role and molecular mechanism of mir-30a-5p in CCA through up-regulating or inhibiting the expression of mir-30a-5p *in vitro* experiments. Together, our study found that mir-30a-5p contributed to the proliferation of CCA cells by directly binding to the downstream target gene SOCS3.

## Materials and methods

### Clinical specimens

From 2015 to 2018, 83 patients underwent surgical resection and pathologically proven CCA (31 ICC and 52 ECC patients respectively) in the First Affiliated Hospital of Nanjing Medical University, China, were included in current study. Inclusion criteria were: Patients with primary diagnosis of CCA between 2015 and 2018, which underwent surgical resection and pathologically proven CCA. Exclusion criteria were: Patients who received neoadjuvant treatment before primary surgery were excluded. The patients combine with hepatocellular carcinoma or other tumors. All specimens were immediately placed in 4% formaldehyde and RNA later protective solution for further study. All samples were collected after obtaining written informed consent from all patients. The study has been approved by the ethics committee of The First Affiliated Hospital of Nanjing Medical University.

### Cell lines culture and transfection

Four human CCA cell lines (HCCC9810, QBC939, RBE, HUCCT1) and normal human bile duct cell line (HiBEC) were obtained from the Cell Bank of the Chinese Academy of Science (Shanghai, China). The cell lines were cultured in Dulbecco's Modified Eagle's Medium (DMEM) containing 10% fetal bovine serum (FBS) and 1% penicillin-streptomycin (all from Winsent Inc., St.Bruno, Quebec, Canada) in a 5% CO_2_ humidified incubator at 37 °C. In order to investigate the role of miR-30a-5p in CCA, inhibition and over-expression of miR-30a-5p were induced by miR-30a-5p agomir and miR-30a-5p antagomir respectively. The transfection was conducted using lipofectamine 2000 (Invitrogen) according to the manufacturer's protocol.

### Cell viability and colony formation assays

Cells were seeded at 1000 cells/well in 96-well plates. Cell viability was measured via Cell Counting Kit-8 (CCK-8, Dojindo, Tokyo, Japan) assay at 0, 24, 48, 72 and 96 hours in terms of the manufacturer's instructions. Absorbance was detected at 450 nm by the spectrophotometer (Thermo Scientific, Pittsburgh, PA, USA). 1×10^4^ cells were seeded in 6-well plates and cell proliferation was measured by the colony-formation assay. Each well was fixed with methanol for 15 min and stained with 0.5% crystal violet for 30 min.

### Quantitative real-time PCR

Total RNA was extracted from CCA tissues and cells with the use of TRIzol reagent (Invitrogen Corp.) The primer sequences involved were as follows: MiR-30a-5p primer-F, 5'-CGCGATGTTGAAACATCCTCGAC-3'; MiR-30a-5p primer-R, 5'-ATCCAGTGCAGGGTCCGAGG-3'; MiR-30a-5p RT primer, 5'-GTCGTATCCAGTGCAGGGTCCGAGGTATTCGCACTGGATACGACCTTCCA-3'. SOCS3 primer-F, 5'-TCGCCACCTACTGAACCCT; SOCS3-3' primer-R, 5'-GGTCCAGGAACTCCCGAAT-3'; The details of RT-PCR were performed according to the manufacturer's instructions.

### Western blot

A protein extraction reagent kit (Beyotime, Shanghai, China) was utilized to extract total protein from human CCA tissues and cells according to the manufacturer's instructions. Western blotting was done with a modified version of a previous method 17. Anti-SOCS3 antibody was purchased from ABCAM. The NIH Image J software (National Institutes of Health, Bethesda, MD) was used to make the results visualized. GAPDH was used as an internal loading control.

### Apoptosis assay

The apoptosis assay was performed by using a FITC annexinV apoptosis detection kit with PI (BioLegend, San Diego, CA, USA). The apoptosis assay was examined by a flow cytometry (FACS Calibur; Becton Dickinson, Franklin Lakes,NJ, USA).

### Immunohistochemical staining

The expression of SOCS3 in CCA tissues were detected by immunohistochemical staining (IHC). The details of IHC staining have been described in our previous article 17. The images were acquired and quantified by light microscopy and NIS-Elements v4.0 software (Nikon, Tokyo, Japan).

### Dual- Luciferase reporter assay

Cells were seeded in 96-well plates and cultured overnight. Luciferase plasmids encoding wild-type or mutant h-SOCS3-3UTR were co-transfected with miR-30a-5p mimics or negative control in terms of the manufacturer's instruction. The luciferase activities were evaluated at 48 h after transfection using the Luciferase Reporter System (Promega, Madison, WI, USA) in terms of the manufacturer's instruction.

### Subcutaneous xenograft

All animal experiments were approved by approved by the ethics committee of The First Affiliated Hospital of Nanjing Medical University. A total of 10 athymic nude mice (4-6 weeks old) were purchased from Model Animal Research Center of Nanjing University, Nanjing, China. CCA cells (2×10^6^) transfected with lentivirus were injected subcutaneously into the flanks of mice to generate xenograft tumors. During the following 4 weeks, tumor size was evaluated every week to plot the tumor growth curve after injections. Tumor volume was calculated with the following formula: Volume=(W^2^× L)/2,where W means tumor width and L means tumor length.

### Statistical analyses

The significance of differences between groups was evaluated with Student's t-test and χ2 test as appropriate. Two-sided p-values were calculated, and a probability level of less than 0.05 was considered to be statistically significant. All statistical data were carried out using SPSS computer software version 16 (SPSS Inc, Chicago, IL, USA).

## Results

### miR-30a-5p was up-regulated in CCA tissues and cells

**Figure 1:**
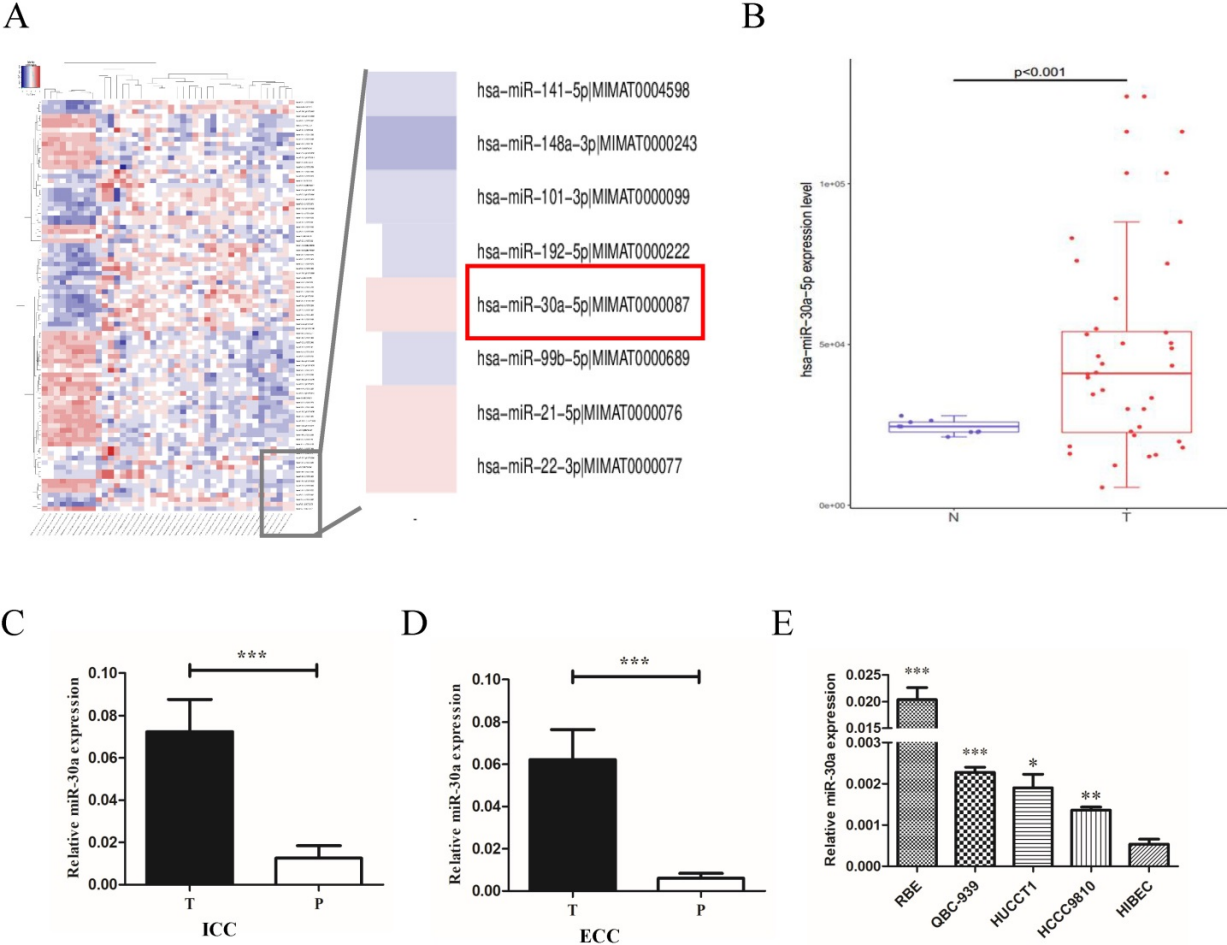
** miR-30a-5p was up-regulated in CCA tissues and cells. (A-B)** miR-30a-5p was up-regulated in CCA tumor tissues compared to para-tumor in public TCGA database. **(C-D)** The expression of miR-30a-5p was over-expressed in 31 paired ICC and 52 paired ECC tumor tissues respectively compared with their corresponding para-tumor tissues. **(E)** The expression of miR-30a-5p was elevated in CCA cells compared to the normal bile duct cells line HiBEC. *p<0.05

To explore the expression profiles of miR-30a-5p in CCA patients, we first examined the expression in CCA patients from public TCGA database. The data showed that compared to para-tumor, miR-30a-5p was up-regulated in CCA tumor tissues (Fig. 1A-B). To confirm the data from public database, miR-30a-5p levels were measured by quantitative RT-PCR in 31 paired ICC and 52 paired ECC samples. Our results showed that the expression of miR-30a-5p was over-expressed in ICC and ECC tumor tissues respectively compared with their corresponding para-tumor tissues (Fig.1C-D). Furthermore, we also detected the expression profiles of miR-30a-5p in CCA cells. The data showed that the expression of miR-30a-5p was elevated in CCA cell lines compared to the normal bile duct cells line HiBEC (Fig. 1E). Collectively, these results suggested that miR-30a-5p was high expression in both human CCA tissues and cells.

### miR-30a-5p promoted CCA cells proliferation

**Figure 2:**
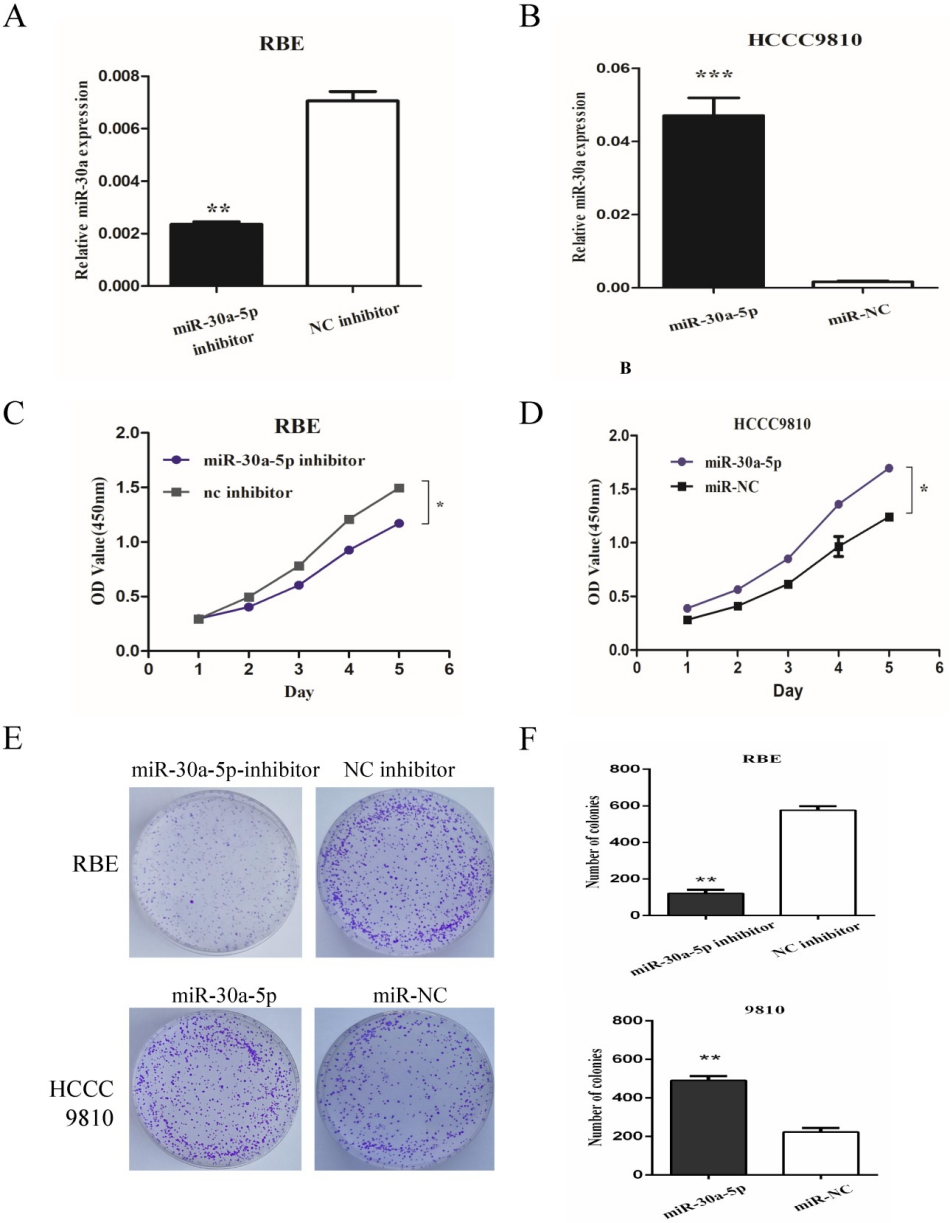
** miR-30a-5p promoted CCA cells proliferation. (A-B)** miR-30a-5p antagomir and agomir were used to inhibit miR-30a-5p in REB cells and up-regulate miR-30a-5p in HCCC9810 cells respectively. **(C-D)** The inhibition of miR-30a-5p significantly inhibited the proliferation of CCA cells while the over-expression of miR-30a-5p promoted the cell proliferation. **(E-F)** The up-regulation of miR-30a-5p promoted the colony formation compared to control group. In contrast, the colony number and size were significantly lower in miR-30a-5p inhibited group. *p<0.05

To determine the role of miR-30a-5p in CCA cells, we used miR-30a-5p antagomir and agomir to inhibit miR-30a-5p in REB cell line (with relatively high miR-30a-5p level) and up-regulate miR-30a-5p in HCCC9810 cell line (with relatively low miR-30a-5p level) respectively (Fig. 2A-B). The results showed that the inhibition of miR-30a-5p significantly inhibited the proliferation of CCA cells while the over-expression of miR-30a-5p promoted the cell proliferation (Fig. 2C-D). Moreover, we also detected the role of miR-30a-5p in cell colony formation. Our data showed that compared to control group, the up-regulation of miR-30a-5p promoted the colony formation. In contrast, the colony number and size were significantly lower in miR-30a-5p inhibited group (Fig. 2E-F). These data indicated that the miR-30a-5p contributed to CCA cells proliferation.

### miR-30a-5p inhibited the apoptosis and promoted tumorigenicity of CAA cells

**Figure 3:**
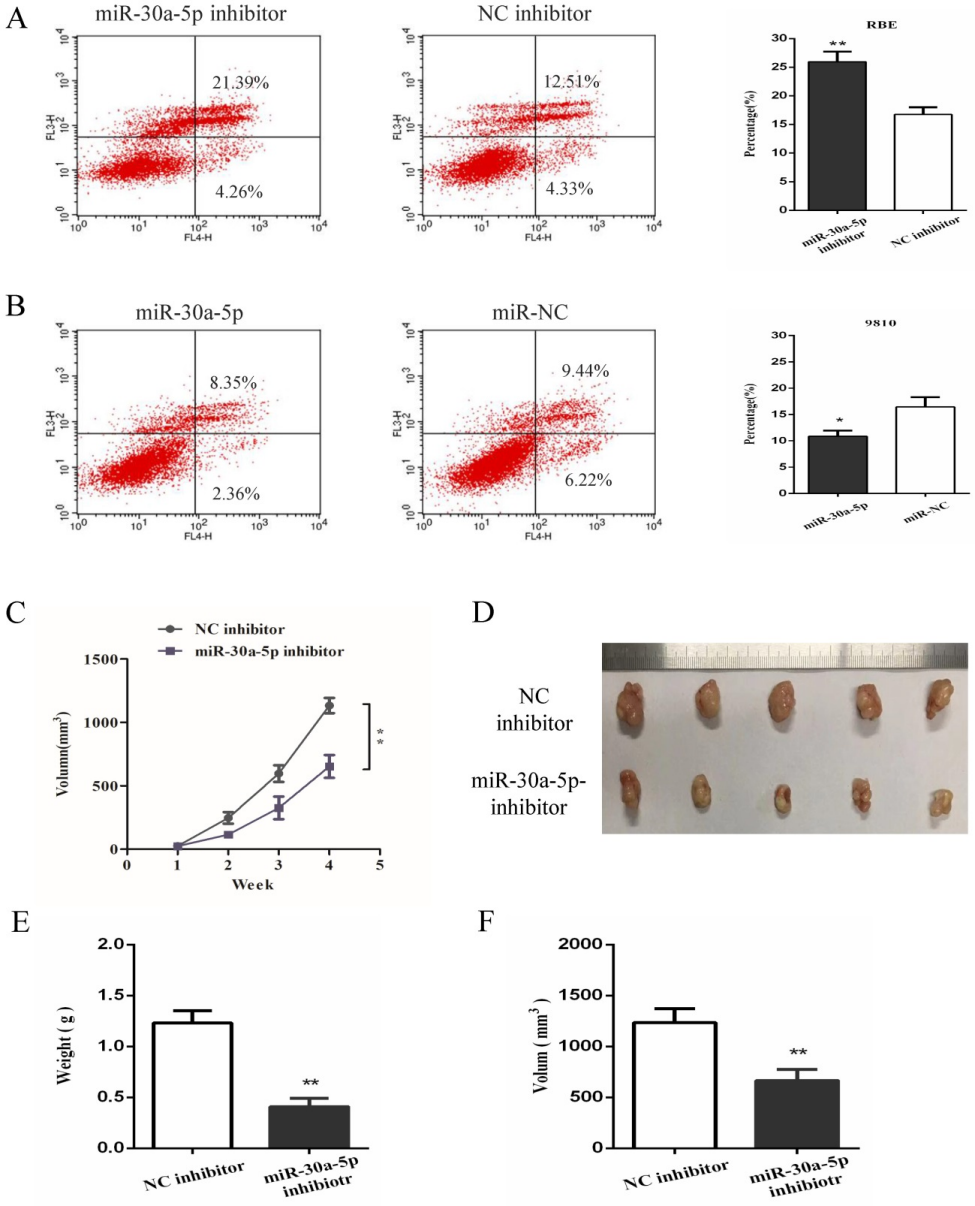
** miR-30a-5p inhibited the apoptosis and promoted tumorigenicity of CAA cells. (A-B)** The inhibition of miR-30a-5p contributed to the apoptosis of CCA cells while up-regulation of miR-30a-5p inhibited CCA cells apoptosis. **(C-F)** Compared to the control group, the inhibition of miR-30a-5p was able to significantly suppress tumorigenicity resulting in obvious reductions in tumor weight and volume. *p<0.05

In order to further investigate the functional role of miR-30a-5p in CCA, cell apoptosis was detected *in vitro* functional study. The date showed that the inhibition of miR-30a-5p contributed to the apoptosis of CCA cells while up-regulation of miR-30a-5p inhibited CCA cells apoptosis (Fig. 3A-B). To further determine the effect of miR-30a-5p on tumorigenicity, xenograft tumor model in nude mice was applied. Our results showed that compared to the control group, the inhibition of miR-30a-5p was able to significantly suppress tumorigenicity resulting in obvious reductions in tumor weight and volume (Fig. 3C-F). These data suggested that miR-30a-5p inhibited CCA cells apoptosis and promoted CCA tumorigenicity.

### SOCS3 was down-regulated in CCA tissues and cell lines

**Figure 4:**
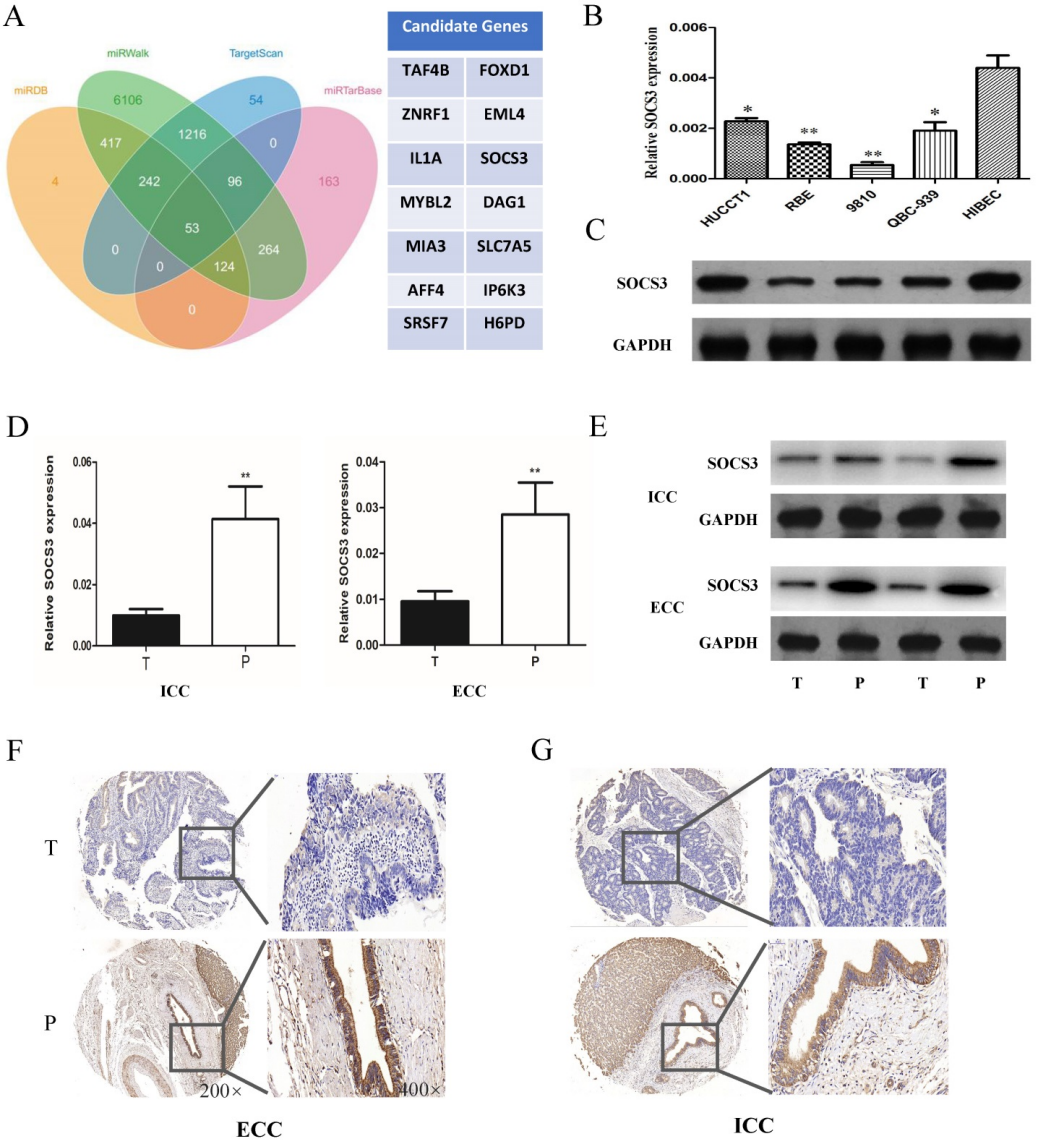
** SOCS3 was down-regulated in CCA tissues and cells. (A)** The biological predicted software predicted the potential target genes of miR-30a-5p. **(B-C)** The expression of SOCS3 was down-regulated in CCA cells compared to the normal bile duct cell line HiBEC. **(D-E)** The expression of SOCS3 was decreased in ICC and ECC tumor tissues respectively compared with their corresponding para-tumor tissues. **(F-G)** IHC staining results confirmed that the expression of SOCS3 was down-regulated in ICC and ECC tissue samples. *p<0.05

To further explore the mechanism of miR-30a-5p in promoting CCA cells proliferation and tumorigenicity, we used the biological predict software to predict the potential target genes of miR-30a-5p (Fig. 4A). The predict results showed that there were about 63 candidate genes of miR-30a-5p. Among of them, SOCS3 is an important negative feedback regulator in the JAK/STAT pathway. The expression of SOCS3 was further confirmed in CCA samples and cell lines. The results showed that the expression of SOCS3 was down-regulated in CCA cells compared to the normal bile duct cell line HiBEC (Fig. 4B-C). In addition, we further validated the results in the clinical tumor tissues from ICC and ECC patients. Our results showed that the expression of SOCS3 was decreased in ICC and ECC tumor tissues respectively compared with their corresponding para-tumor tissues (Fig. 4D-E). IHC staining results also confirmed that the expression of SOCS3 was down-regulated in ICC and ECC tissue samples (Fig. 4F-G). These data suggested that the SOCS3 maybe a suppressor gene of CCA.

### miR-30a-5p negatively regulated the expression of SOCS3

**Figure 5:**
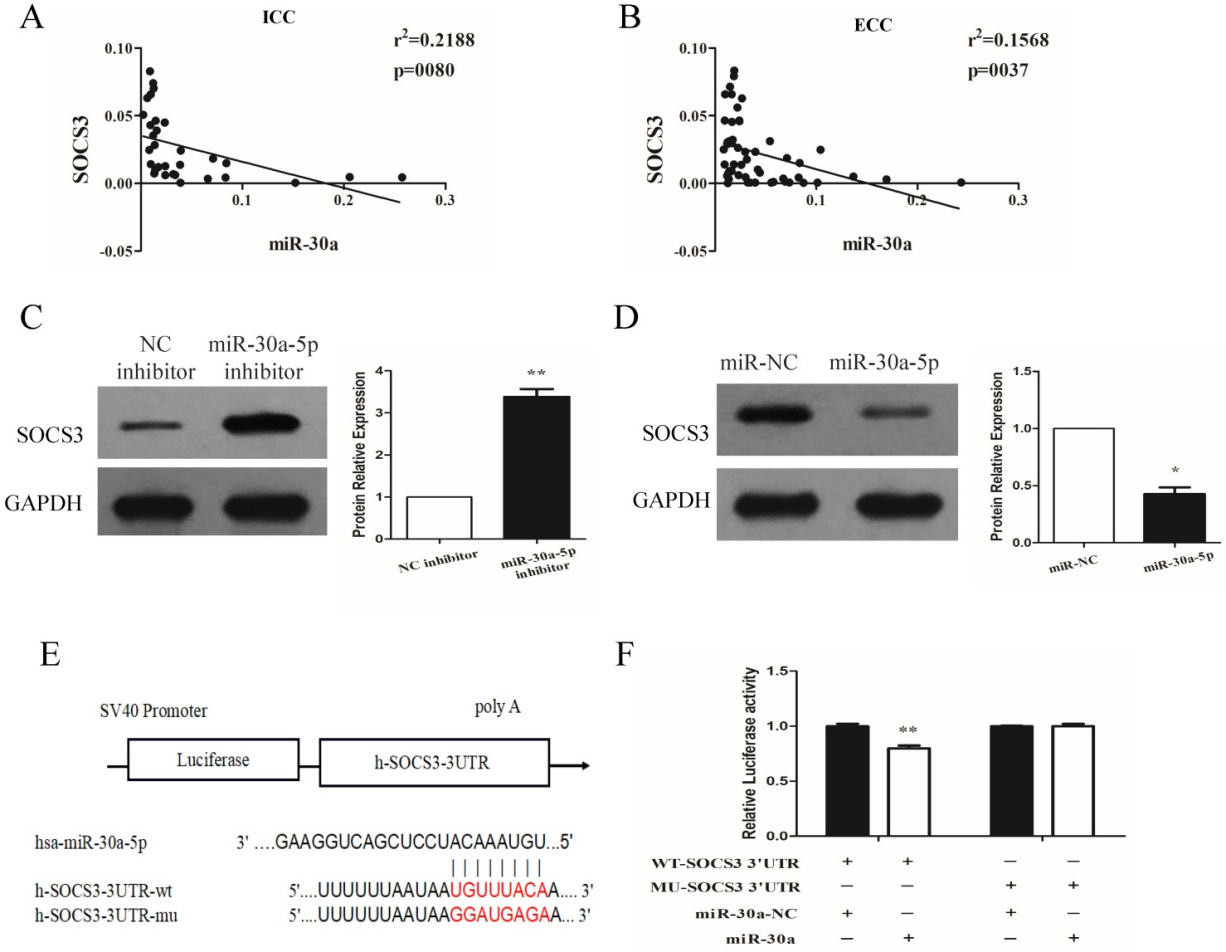
** miR-30a-5p negatively regulated the expression of SOCS3. (A-B)** The expression of SOCS3 was negatively correlated with miR-30a-5p expression in ICC and ECC tissue samples. **(C)** The inhibition of miR-30a-5p increased the expression of SOCS3. **(D)** The expression of SOCS3 was significantly decreased in miR-30a-5p up-regulation group. **(E)** 3′UTR of SOCS3 exhibited a putative binding site of miR-30a-5p. **(F)** Dual-luciferase reporter assay revealed that co-transfection of miR-30a-5p significantly inhibited the activity of firefly luciferase reporter carrying the wild-type 3′UTR of SOCS3. *p<0.05

We next investigated the expression relationship between miR-30a-5p and SOCS3 in CCA samples. Our data showed that the expression of SOCS3 was negatively correlated with miR-30a-5p expression in ICC and ECC tissue samples (Fig. 5A-B). These results were further confirmed in CCA cell lines. The results showed that compared to control group, the inhibition of miR-30a-5p increased the expression of SOCS3. In addition, we also found that the expression of SOCS3 was significantly decreased in miR-30a-5p up-regulation group (Fig. 5C-D). After detailed sequence analysis, we observed that 3′UTR of SOCS3 exhibited a putative binding site of miR-30a-5p (Fig. 5E). Dual-luciferase reporter assay revealed that co-transfection of miR-30a-5p significantly inhibited the activity of firefly luciferase reporter carrying the wild-type 3′UTR of SOCS3 (Fig. 5F). These data suggested that SOCS3 can be negatively regulated by miR-30a-5p in CCA cells.

### miR-30a-5p regulated proliferation of CAA cells in a SOCS3 dependent manner

**Figure 6:**
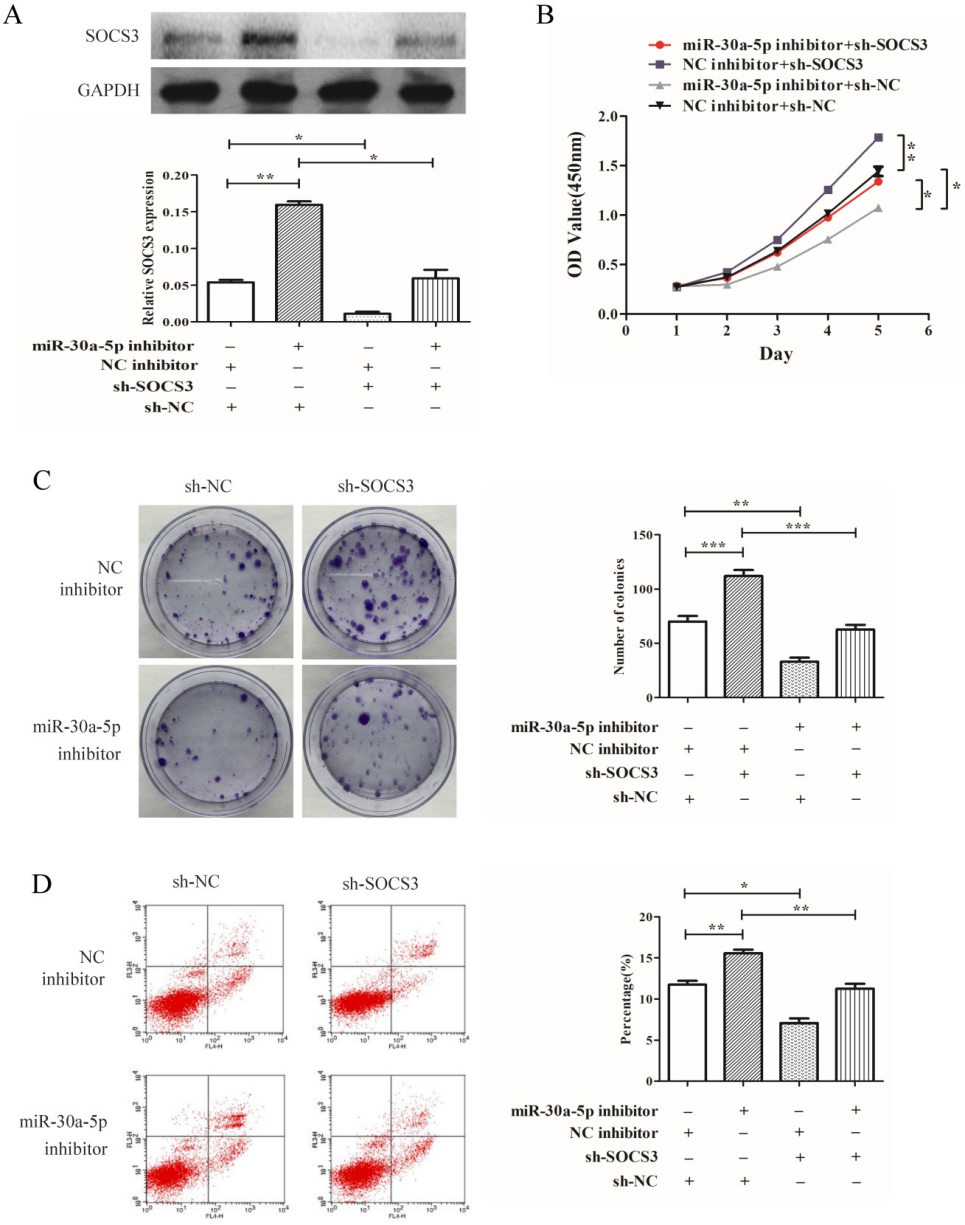
** miR-30a-5p regulated proliferation of CAA cells in a SOCS3 dependent manner. (A)** The expression of SOCS3 was inhibited by shRNA. **(B)** Compared to miR-30a-5p inhibition alone, the inhibition of SOCS3 could rescue the inhibitory effect of miR-30a-5p inhibition on proliferation of CCA cells. **(C) (B)** The inhibition of SOCS3 could rescue the inhibitory effect of miR-30a-5p inhibition on colony formation in CCA cells. **(D)** The inhibition of SOCS3 significantly suppressed cells apoptosis compared to the control group. *p<0.05

To determine whether SOCS3 was key mediator of miR-30a-5p's function in cellular proliferation, SOCS3 was inhibited by shRNA (Fig. 6A). Our results demonstrated that the inhibition of SOCS3 promoted the CCA cells proliferation and colony formation. More importantly, compared to miR-30a-5p inhibition alone, the inhibition of SOCS3 could largely rescue the inhibitory effect of miR-30a-5p inhibition on proliferation and colony formation in CCA cells line (Fig. 6B-C). Furthermore, the inhibition of SOCS3 significantly suppressed cells apoptosis compared to the control group (Fig. 6D). These results indicated that miR-30a-5p regulated proliferation of CAA cell in a SOCS3 dependent manner.

### The clinical significance of miR-30a-5p in CCA patients

To explore the clinical significance of miR-30a-5p in CCA patients, the correlation of miR-30a-5p expression with clinicopathological parameters of ICC and ECC were analyzed respectively. The results demonstrated that over-expression of miR-30a-5p was positively associated with large tumor size and tumor nodules in ICC cohorts (Table 1). Furthermore, the expression of miR-30a-5p was correlated with tumor size in ECC cohorts (Table 2). Our results also showed that the expression of SOCS3 was negatively correlated with miR-30a-5p expression in ICC and ECC tissue samples. These results collectively suggested that up-regulated miR-30a-5p and lower SOCS3 expressions might have a stimulatory role in the progression of CCA patients.

## Discussion

To our knowledge, this is the first report on the roles and mechanisms of miR-30a-5p in CCA. In this study, our results demonstrated that the expression of miR-30a-5p was increased in both CCA tissues and cells. It was consistent with previous data displayed in the TCGA database. Furthermore, overexpression of miR-30a-5p was significantly associated with tumor size and nodules of CCA patients. The inhibition of miR-30a-5p suppressed tumor cell proliferation and induced cell apoptosis. Collectively, these data suggested that miR-30a-5p could be a potential therapeutic target of CCA.

miR-30a-5p, as a member of the mir-30 family, plays a crucial role in tumor growth and metastasis 5, 6. Currently, the expression profile and function of miR-30a-5p in different malignancies are still existed controversies. A large number of reports have demonstrated that miR-30a-5p was commonly down-expression and regulated biological behaviors of tumor growth, invasion, epithelial-mesenchymal transition (EMT) in various types of cancers 13, 14, 18. miR-30a-5p was down-regulated in prostate cancer and inhibited cell proliferation via targeting PCLAF 14. Furthermore, the down-regulation of miR-30a-5p was associated with poor prognosis and promoted chemoresistance of gemcitabine in pancreatic ductal adenocarcinoma 19. However, it has been also reported that overexpression of miR-30a-5p contributed to initiation and progression of some tumors^12^, ^16^. miR-30a-5p was increased and as a potential biomarker for ovarian serous adenocarcinoma^16^. In addition, the expression of miR-30a-5p was increased in glioma tissues and promoted glioma cell proliferation, migration, and invasion via targeting WWP1 12. These consistent with our present data. miR-30a-5p was over-expressed in CCA samples and cells. The inhibition of miR-30a-5p suppressed tumor cell proliferation and induced cell apoptosis of CCA.

Suppressor of cytokine signaling (SOCS) proteins, as negative feedback regulators of cytokine signaling, are induced by interleukins and various peptide hormones and may prevent sustained activation of signaling pathways 20. SOCS3, a member of SOCS family, acts as a negative feedback regulator of the Janus‐activated kinase/signal transducers and activators of transcription factor (JAK/STAT) signaling pathway 21, 22. More evidence showed that abnormal expression of SOCS3 is observed in a variety of malignant tumors, which may lead to promoter methylation and further contribute to the occurrence and development of tumors 23, 24. Hajime et al showed that reconstitution of SOCS3 expression reduced the duration and magnitude of IL-6-mediated STAT-3 phosphorylation, decreased cellular Mcl-1 levels, and sensitized CCA cells to TRAIL-induced killing 25. Furthermore, overexpression of SOCS3 inhibited the IL-6-induced epithelial-to-mesenchymal transition and CCA cell metastasis 26. Recently, increasing research showed that microRNA regulated tumor development and progression through targeting SOCS expression^27^, ^28^. However, whether miR-30a-5p promoted CCA cells proliferation by regulating SOCS3 has not been reported. In this study, the expression of SOCS3 was up-regulated in miR-30a-5p -inhibition CCA cell lines. Furthermore, the IHC results in CCA patients showed that there was significantly negatively association between miR-30a-5p with SOCS3. More interesting, the inhibition of SOCS3 could largely rescue the inhibitory effect of miR-30a-5p inhibition on proliferation of CCA cells. Dual-luciferase reporter assay revealed that co-transfection of miR-30a-5p significantly inhibited the activity of firefly luciferase reporter carrying the wild-type 3′UTR of SOCS3. These data demonstrated that miR-30a-5p negatively regulated the SOCS3 signaling to exert its tumor-promoted functions in CCA.

In summary, we demonstrated that miR-30a-5p was up-regulation and significantly associated with tumor size and nodules of CCA patients. The inhibition of miR-30a-5p decreased cell proliferation and induced cell apoptosis while overexpression of miR-30a-5p achieved the opposite effect. Furthermore, we also showed that miR-30a-5p promoted CCA progression through regulating SOCS3 signaling pathway. Together, miR-30a-5p could be a potential therapeutic target for CCA. A larger sample size is needed to further explore the clinical significance of miR-30a-5p in CCA patients.

## Figures and Tables

**Table 1:** The correlation of miR-30a-5p expression with clinicopathological parameters in ICC patients

ICC Clinicopathologic Features	n	miR-30a-5p		
		high	low	P
**Gender**				
Female	12	6	6	0.886
Male	19	9	10	
**Age**				
<60	16	7	9	0.594
≥ 60	15	8	7	
**No. of tumor nodules**				
1	18	5	13	0.022*
>1	13	9	4	
**Tumor size**				
< 5	11	3	8	0.022*
≥ 5	20	14	6	
**Pathological stages**				
I + II	9	4	5	0.779
III + IV	22	11	11	
**Vascular invasion**				
Absent	19	11	8	0.625
Present	12	8	4	
**Tumor Stages**				
I + II	16	10	6	0.632
III + IV	15	7	8	

**Table 2:** The correlation of miR-30a-5p expression with clinicopathological parameters in ECC patients

ECC Clinicopathologic Features	n	miR-30a-5p		
		high	low	P
**Gender**				
Female	16	6	10	0.309
Male	36	19	17	
**Age**				
<60	34	17	17	0.703
≥ 60	18	8	10	
**Tumor size**				
< 3	32	13	19	0.016*
≥ 3	20	15	5	
**Pathological stages**				
I + II	23	11	12	0.473
III + IV	29	11	18	
**Vascular invasion**				
Absent	37	11	26	0.110
Present	15	8	7	
**Tumor Stages**				
I + II	25	10	15	0.262
III + IV	27	15	12	

## Abbreviations

SOCS): MicroRNAs (miRNAs
